# Comparative study of armchair and zigzag graphene quantum dots as HIV-1 protease inhibitors

**DOI:** 10.1038/s41598-026-48709-7

**Published:** 2026-05-07

**Authors:** Asmaa Ibrahim, Hanan Elhaes, Medhat A. Ibrahim

**Affiliations:** 1 Physics Department, Faculty of Women for Arts, Science and Education, Ain Shams University, Cairo, 11757 Egypt; 2 Spectroscopy Department, National Research Centre, 33 El-Bohouth St., Dokki, Giza, 12622 Egypt; 3 Center for Converging Sciences and Emerging Technologies (CoSET), Benha National University (BNU), El-Obour, 13518 Egypt; 4 Molecular Modeling and Spectroscopy Laboratory, Centre of Excellence for Advanced Science, National Research Centre, 33 El-Bohouth St., Dokki, Giza, 12622 Egypt

**Keywords:** DFT, B3LYP/6-31G(d, p), Graphene quantum dots GQDs and HIV-1 protease inhibitors, Chemistry, Materials science, Nanoscience and technology, Physics

## Abstract

In this study, five distinct types of graphene quantum dots (GQDs) which included pristine graphene and three types of triangular cuts which had both armchair and zigzag ends and two types of hexagonal cuts which had both armchair and zigzag ends. Density functional theory (DFT) with the B3LYP/6-31G(d, p) method was used to determine total dipole moment (TDM) values and to analyze HOMO-LUMO energy gaps and molecular electrostatic potential (MESP) and the vibrational spectra of the researched models. The study utilized quantum theory of atoms in molecules (QTAIM) analysis to assess the bonding relationships between the atoms. The results demonstrate that the modified GQDs can bind with two aspartic acid residues to create stable complexes. The study demonstrates that modified GQDs can function as potential candidates for use as HIV-1 protease inhibitors.

## Introduction

Graphene is a carbon-based material that can be described as a two-dimensional (2D) material formed of thin carbon atoms assembled in a honeycomb structure. It was isolated for the first time in 2004 following the mechanical exfoliation of graphite^1^. Physically, graphene features an electronic band structure characterized by a massless Dirac spectrum, leading to distinct physical properties^2,3^. These features dedicate graphene to enormous applications such as spintronic, photonic, and optoelectronic^4–6^. Based on its zero energy band gap, it could be applied effectively as a semiconductor device^7,8^. Another form of graphene is widely applied in several fields named graphene quantum dots (GQDs), this type of graphene shows confined charges in all directions, which in turn enhances the application of this kind of graphene in several electronic applications^9^. GQDs have various shapes and edge terminations that can be chemically functionalized^10–12^ to have numerous applications, not limited to electronic applications but also including biomedical applications^13,14^. Besides the exceptional electronic, thermal, and mechanical stability of graphene, GQDs possess both quantum confinement and edge effects^15^. Moreover, the existence of sp2-sp2 carbon bonds beside high electron mobility, adjustable band gaps, favorable biocompatibility, and effective solubility in different solvents. GQDs show the potential applications in biomedical fields such as drug delivery, biosensing and bioimaging^16–18^. Chemical functionalization of GQDs tunes its characteristics, functional groups, especially those containing oxygen, significantly enhance the solubility and biological activity of GQDs as stated earlier^19,20^. Further enhancement could be achieved as GQDs are treated chemically with elements having an identical atomic radius, because of their ease of bonding with carbon^21^. The GQDs show potential applications for cellular imaging of the MCF-7 human breast cancer. It has been stated that they exhibit fluorescence with low cytotoxicity, allowing for the labeling of the cell membrane, cytoplasm, and nucleus. This in turn initiates the so-called cellular imaging and diagnosis^22^. Later on, some researchers apply functionalized GQDs as an effective tool in bioimaging applications^23,24^. The GQDs loaded with natural materials show therapeutic activity, for example when it loaded with curcumin it exhibited anticancer activity^25^. Modified GQDs show also dual functions as it could be used to deliver and also to treat^26^. It is stated that, modified GQDs could replace traditional chemotherapy as the modified GQDs ligand-based drugs within nanomaterials to act with dual functions^27^. According to the unique physicochemical and biological characteristics of modified GQDs, many researchers apply modified GQDs as a promising tools in therapeutic applications^28–30^. Modified GQDs are dedicated to overcoming limitations posed by tumor physiology makes them a promising tool for cancer therapy, according to their unique small size which allow them to traverse cellular membranes, enhancing the drug accumulation and permeability in the targeted tissues^31,32^. Carbon based materials specially in nano form show an excellent photodynamic antibacterial activity for treatment of bacterial infections^33,34^. The structural, stability, and electronic characteristics of ZnO- GQDs in various locations was reported. Results dedicated the modified to GQDs nanoelectronics, biosensors, and nanomedicine applications^35^. Later on, it was found that, the electronic properties of GQDs can be altered through metalloid doping such as boron (B), silicon (Si), germanium (Ge), and arsenic (As)^36^. It was reported that, heteroatom doping of GQDs show antiviral properties^37^. For example the modification of GQDs with nitrogen can be used to combat two kinds of viruses including Feline Coronavirus NTU156 (FCoV NTU156) and Enterovirus 71 (EV71)^38^.

The functionalized graphene quantum dots GQDs show strong potential to function as inhibitors because of their ability to stay bound and their capacity to detect electronic changes. The hydrogen-saturated zig-zag edges serve as a dependable basis which researchers use to study how molecules get trapped on graphene materials with limited surface area^39^. The ZTRI/Pyrrolidine/2HMC complex interacts with aspartic acid residues which results in observable changes to both the HOMO-LUMO energy gap and total dipole moment values which scientists use to assess how molecules absorb onto surfaces^40^. The GQD practical efficiency gets established through their recovery time measurement which scientists denote as($\tau$). According to transition state theory proteins achieve both strong binding capacity and free movement abilities which protect their active sites through short recovery periods at body temperature^40,41^. The armchair-edged graphene nanosheets was studied as bio-sensing platforms by assessing their structural and electronic properties for detecting chitosan and glucose and cholesterol monomers. Through DFT analysis, it was shown that functionalized graphene interacts with biological molecules to produce strong chemical adsorption at its base state, which demonstrates that finite graphene models are useful for studying molecular interactions in biological systems^42^.

Gathering the above data it is clear that the unique properties of GQDs enable it for several applications not limited to the electronic applications but also to biomedical applications paving the way toward further enhancement in GQD modification for further applications. There are four types of GQDs, both ATRI and AHEX are the triangular and hexagonal with armchair termination. While both ZTRI and ZHEX are triangular and hexagonal with zigzag edges^43^.

The aim of the present work is to enhance the structure of five types of GQDs (Pristine graphene, ATRI, ZHEX, ZTRI and AHEX) with Pyrrolidine ring and benzene to carry out two carboxymethyl group HMC. It is a first-principles approach to comprehensively investigate the structural, electronic, and vibrational characteristics of various functionalized Graphene Quantum Dots (GQDs) to assess their potential as novel HIV-1 Protease inhibitors. So that, the proposed structures were optimized with density functional theory DFT: B3LYP/6-31G(d, p) then some physical parameters were calculated at the same level of theory. According to the hydrogen bonding in the two HMC groups, interaction is supposed between them and carboxyl group of two units of aspartic acids. The overall aim is to indicate the ability of the modified GQDs as HIV-1 protease inhibitor.

## Calculations details

**Fig. 1:**
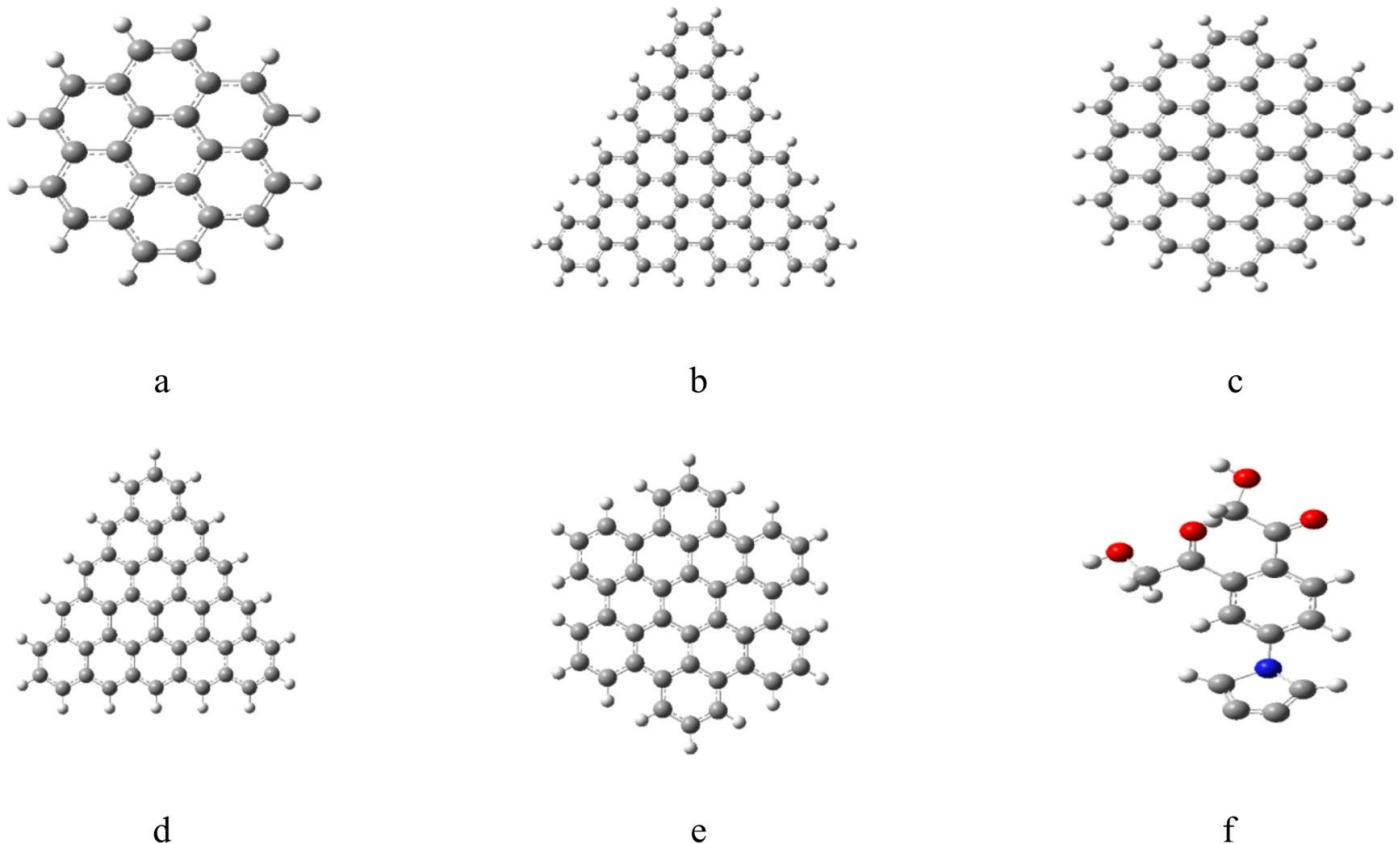
Proposed model molecules for graphene quantum dots (**a**) pristine graphene, (**b**) triangular cuts with armchair ends (ATRI), (**c**) hexagonal cuts with zigzag ends (ZHEX), (**d**) zigzag triangular structure (ZTRI), (**e**) hexagonal cuts with zigzag ends (AHEX) and (**f**) Pyrrolidine/benzene/2HMC which is used to modfiy the each one of the studied graphene quantum dots. Colour coding: Gray: Carbon (C); White: Hydrogen (H); Red: Oxygen (O); Blue: Nitrogen (N).

The graphene model molecules are indicated in Fig. 1, these models represent five types of graphene quantum dots GQDs namely a- pristine graphene, b- triangular cuts with armchair ends (ATRI), c- hexagonal cuts with zigzag ends (ZHEX), d- triangular cuts with armchair ends (ZTRI) and e- hexagonal cuts with zigzag ends (AHEX) respectively. Each type of GQD was further modified with a Pyrrolidine ring, followed by a benzene ring with two HMC groups, as indicated in Fig. 1-f.

The graphene model molecules, representing five types of graphene quantum dots (GQDs), were subjected to geometry optimization and electronic property calculations using the G09 program^44^, which is implemented at the Molecular Modeling and Spectroscopy Laboratory, Centre of Excellence for Advanced Science, National Research Centre, Egypt. First, each structure was subjected to optimization using Density Functional Theory (DFT) at the B3LYP/6-31G(d, p) level^45–47^. For all studied models, a neutral charge (0) and singlet multiplicity (1) were used.

The Total Dipole Moment (TDM) which was calculated as the vector sum of the individual bond dipoles in the optimized molecular configuration.The HOMO-LUMO Energy Gap (ΔE) was defined and calculated using the formula: ΔE = E_LUMO_-E_HOMO_ where E_HOMO_ and E_LUMO_ represent the energies of the highest occupied and lowest unoccupied molecular orbitals, respectively, the molecular electrostatic potential MESP. The B3LYP/6-31G(d, p) level provides a reliable balance between computational cost and accuracy for characterizing the HOMO-LUMO gaps and total dipole moments of similar systems as indicated previously^42^.

Vibrational spectra for the studied models were also calculated at the same level of theory. To assess the stability of the studied structures one important analysis was conducted. The Quantum Theory for Atoms in Molecules (QTAIM) analysis which conducted with the “output = wfn” command in the G09 program, then visualized with the help of Multiwfn and Visual molecular dynamics (VMD) software^48,49^.

## Results and discussions

### Studied physcial parameters

Table 1 presented some physical parameters calculated at B3LYP/6-31G(d, p) such as total dipole moment TDM, HOMO/LUMO energy gap and molecular dimensions. Results indicated that, the Pristine graphene has TDM as 0.0000 Debye and ∆E was 4.0344 eV. For Pristine/Pyrrolidine, the TDM increased to be 3.5179 Debye while the ∆E was decreased to 3.4583 eV.The same behaviour is noticed for Pristine/Pyrrolidine/Benzene/2HMC. For graphene quantum dots ATRI, it has 0.000 TDM and ∆E was 3.1930 eV. As it enhanced to form ATRI/Pyrrolidine the TDM increased to be 4.6908 Debye while the ∆E was decreased to 2.8131 eV. For ATRI/Pyrrolidine/Benzene/2HMC the TDM was also increased to 4.5956 Debye then ∆E was decreased to 2.4651 eV.ZHEX shows TDM 0.0000 Debye, while ∆E was 2.8210 eV. Then it enhanced to form ZHEX/Pyrrolidine with TDM increased to 4.6908 Debye then ∆E was decreased 2.6896 eV. The ZHEX/Pyrrolidine/Benzene/2HMC structure shows TDM as 3.8286 Debye (lower than that for ZHEX/Pyrrolidine, but ∆E was also decreased to 2.4825 eV. ZTRI shows TDM of 0.0021 Debye and ∆E was of 0.3216 eV. TDM was increased up to 7.6579 Debye corresponding to ZTRI/Pyrrolidine, while ∆E was decreased to 0.2884 eV. ZTRI/Pyrrolidine/2HMC, shows TDM equal 6.6474 Debye, and the ∆E was 0.2844 eV. Finally, the AHEX shows TDM as 0.0000 Debye and ∆E was 3.5892 eV.Then TDM was 3.8230 Debye for AHEX/Pyrrolidine, while ∆E was decreased as 2.9821 eV. AHEX/Pyrrolidine/Benzene/2HMCshows further increase in TDM as 5.2611 Debye, the ∆E was decreased to 2.2950 eV.

It was previously stated that structures showing a higher TDM with a lower ∆E may indicate higher reactivity compared to other structures^50,51^. On this basis comparing the results in Table 1 revealed that, the structure ZTRI/Pyrrolidine/2HMC shows higher TDM (6.6474 Debye) with lower ∆E (0.2844 eV) in comparison with other enhanced structures, it could be more reactive with the surrounding molecules. As listed in Table 1, the molecular dimension varies from 9.279 Å corresponding to Pristine/Pyrrolidine/Benzene/2HMC structure up to 17.884 Å corresponding to ATRI/Pyrrolidine/Benzene/2HMC structure.

**Fig. 2:**
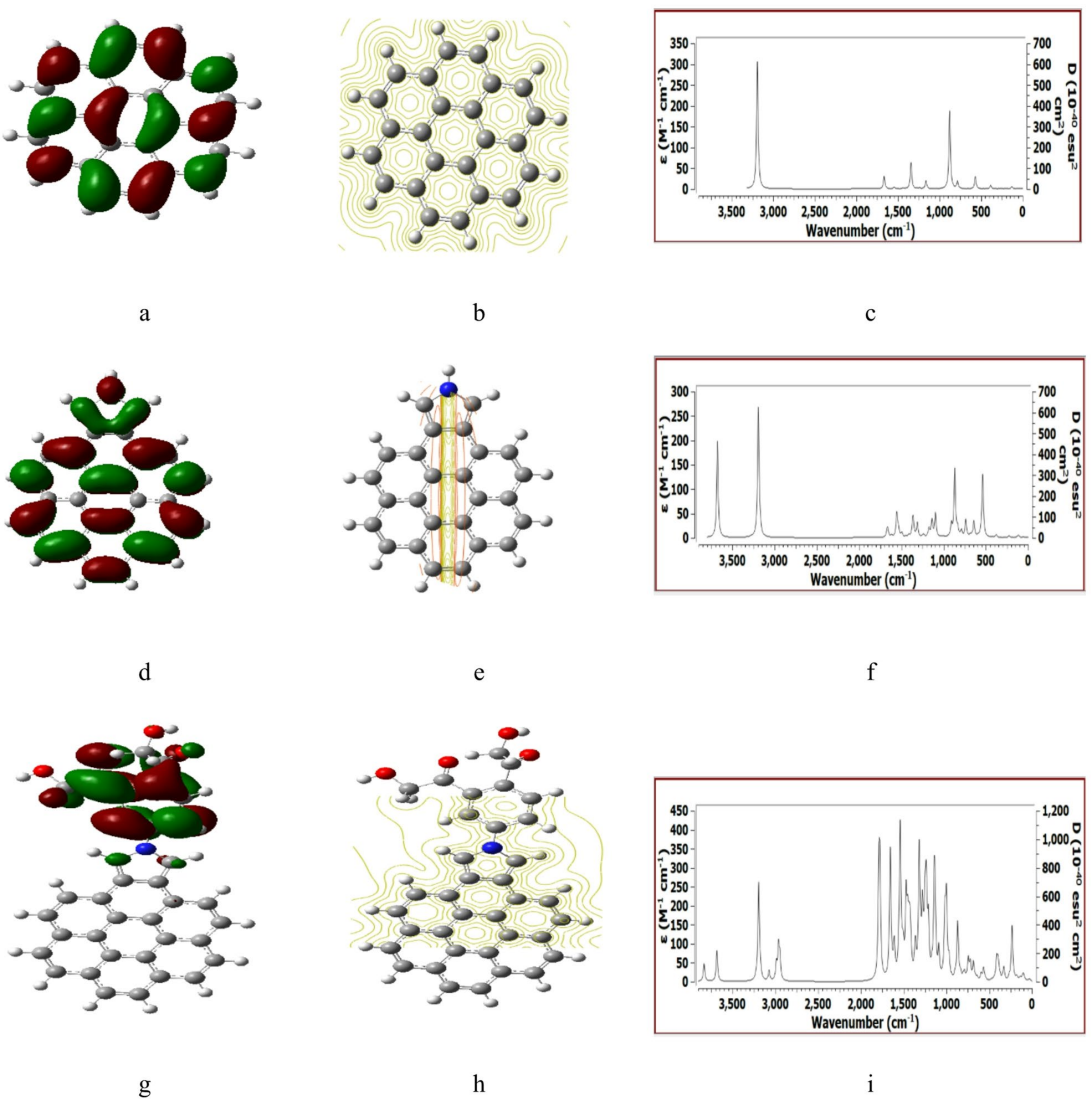
DFT: B3LYP/6-31G(d, p) calculated parameters for Pristine graphene whereas, (**a**) HOMO/LUMO, (**b**) MESP, (**c**) IR spectrum. Pristine graphene/Pyrrolidine (**d**) HOMO/LUMO, (**e**) MESP, (**f**) IR spectrum. Pristine graphene/Pyrrolidine/2HMC (**g**) HOMO/LUMO, (**h**) MESP and (**i**) IR spectrum.

**Fig. 3:**
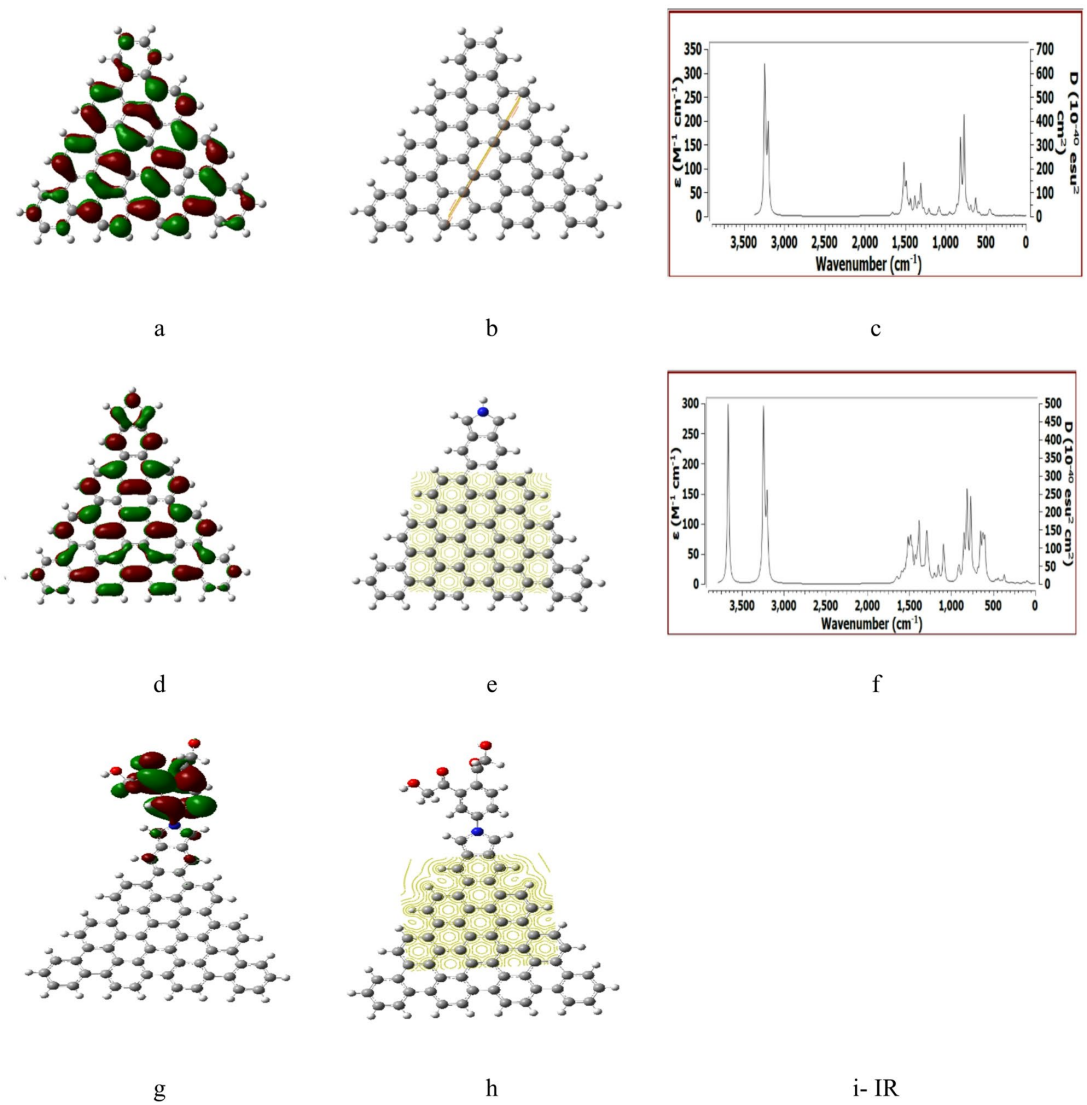
DFT: B3LYP/6-31G(d, p) calculated parameters for ATRI whereas, (**a**) HOMO/LUMO, (**b**) MESP, (**c**) IR spectrum. ATRI/Pyrrolidine (**d**) HOMO/LUMO, (**e**) MESP, (**f**) IR spectrum. ATRI/Pyrrolidine/Benzene/2HMC (**g**) HOMO/LUMO, (**h**) MESP and (**i**) no IR spectrum was found at this level of theory.

**Fig. 4:**
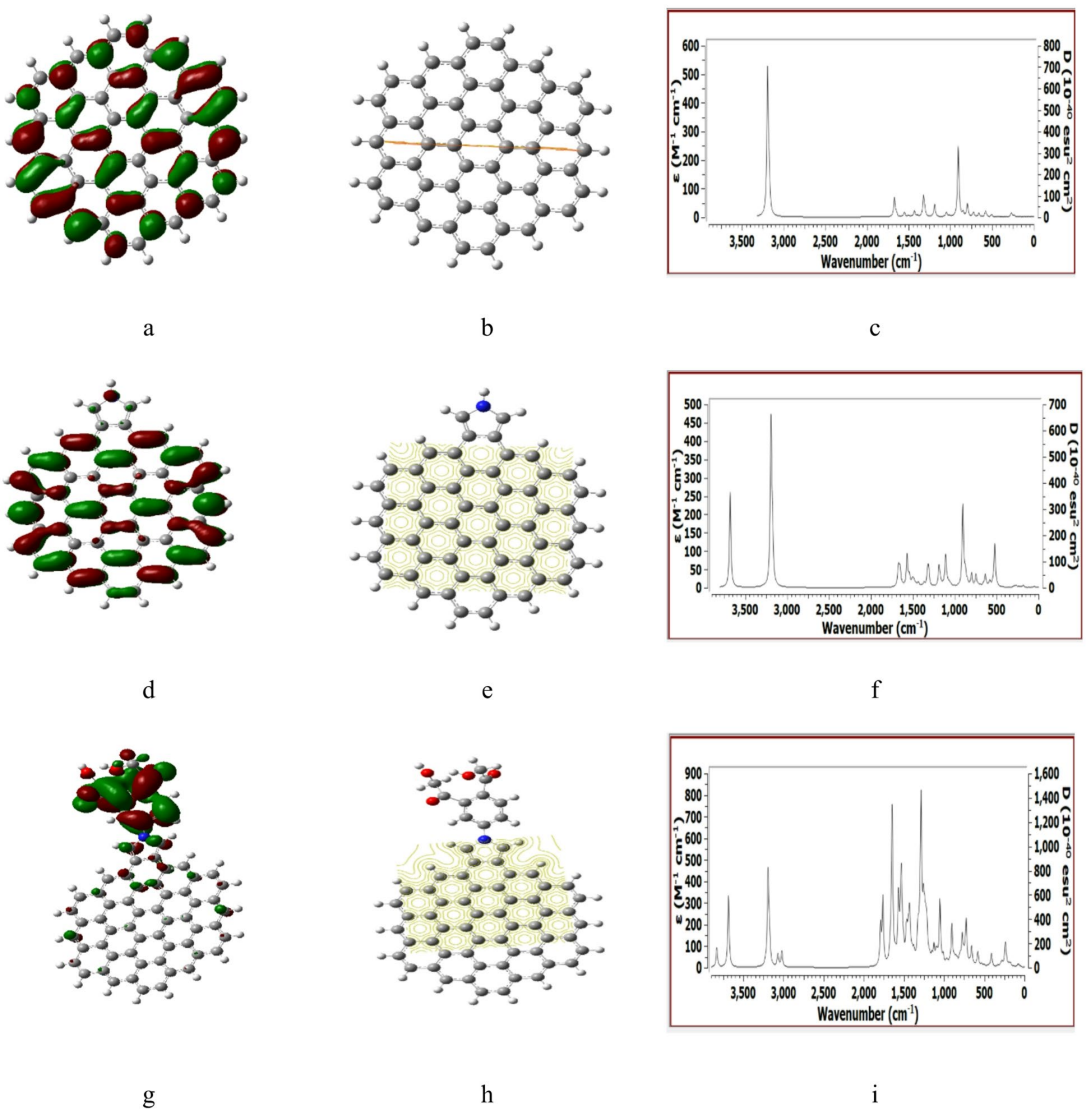
DFT: B3LYP/6-31G(d, p) calculated parameters for ZHEX whereas, (**a**) HOMO/LUMO, (**b**) MESP, (**c**) IR spectrum. ZHEX /Pyrrolidine (**d**) HOMO/LUMO, (**e**) MESP, (**f**) IR spectrum. ZHEX/Pyrrolidine/Benzene/2HMC (**g**) HOMO/LUMO, (**h**) MESP and (**i**)- IR spectrum.

**Fig. 5:**
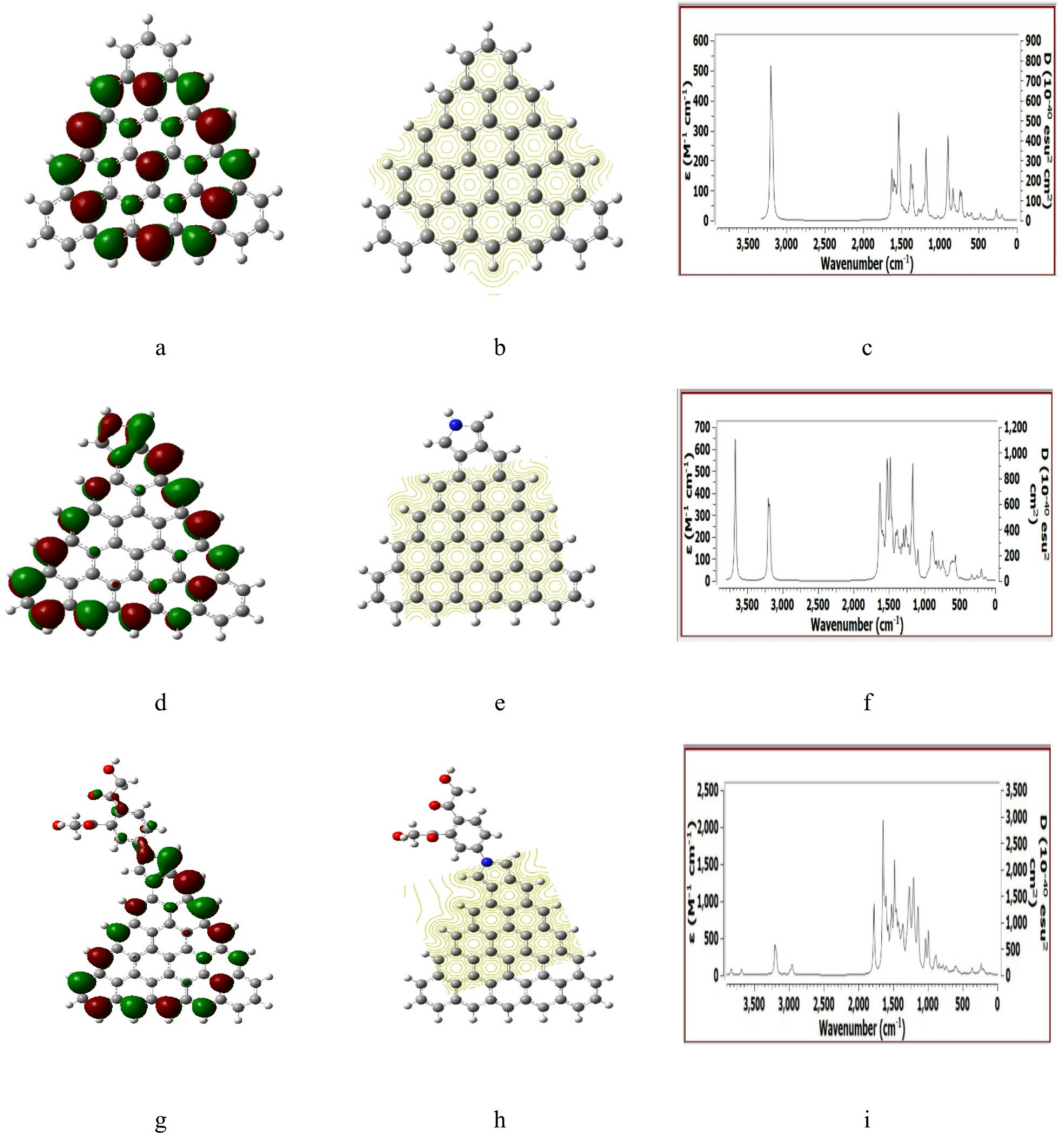
DFT: B3LYP/6-31G(d, p) calculated parameters for ZTRI whereas, (**a**) HOMO/LUMO, (**b**) MESP, (**c**) IR spectrum. ZTRI /Pyrrolidine (**d**) HOMO/LUMO, (**e**) MESP, (**f**) IR spectrum. ZTRI /Pyrrolidine/2HMC (**g**) HOMO/LUMO, (**h**) MESP and (**i**) IR spectrum.

**Fig. 6:**
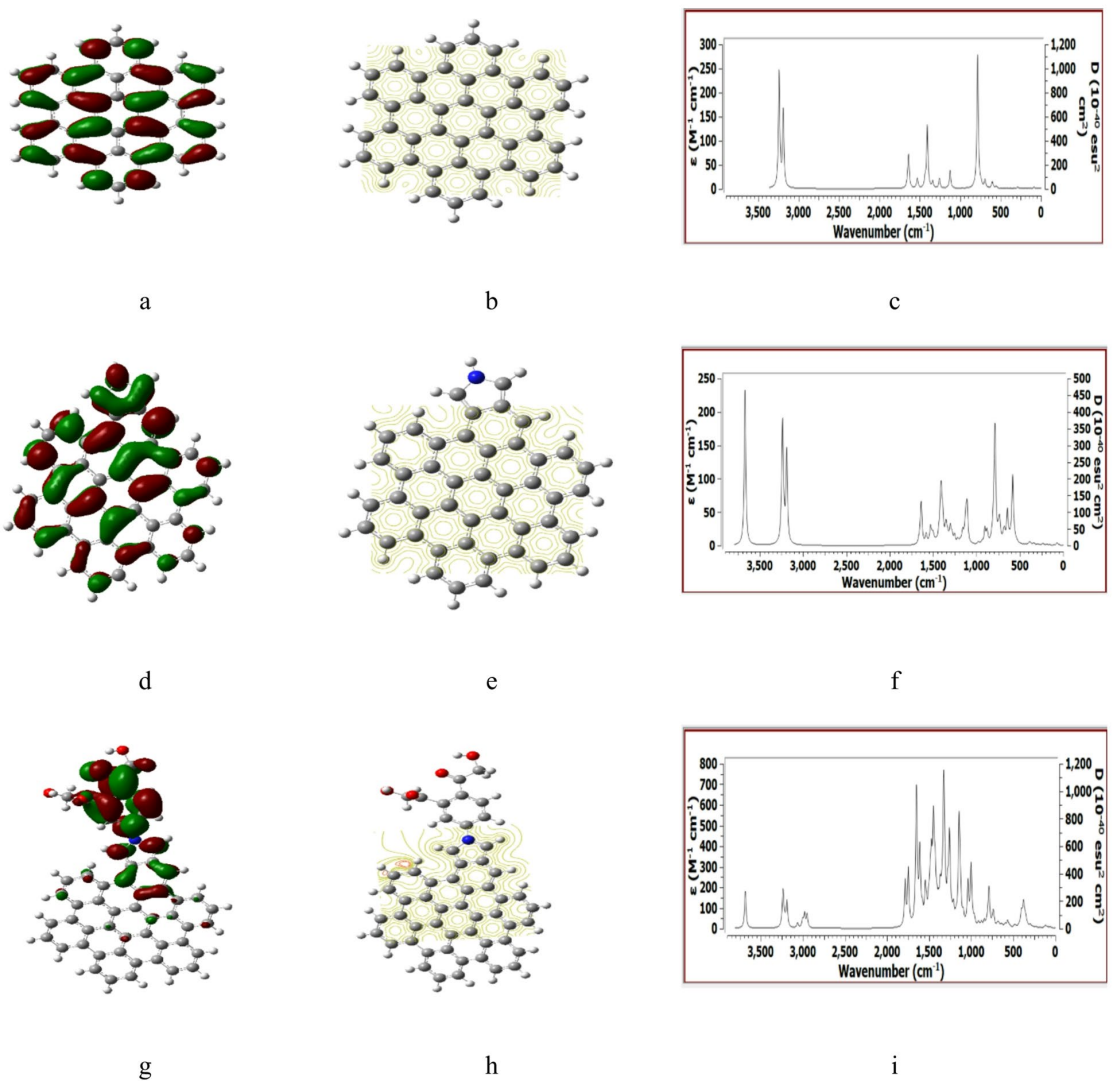
DFT: B3LYP/6-31G(d, p) calculated parameters for AHEX whereas, (**a**) HOMO/LUMO, (**b**) MESP, (**c**) IR spectrum. AHEX/Pyrrolidine (**d**) HOMO/LUMO, (**e**) MESP, (**f**) IR spectrum. AHEX /Pyrrolidine/2HMC (**g**) HOMO/LUMO, (**h**) MESP and (**i**) IR spectrum.

Figures 2, 3, 4, 5 and 6 presented the B3LYP/6-31G(d, p) calculated HOMO/LUMO orbitals, molecular electrostatic potentials MESP, and the IR spectrum for the studied structures.

As indicated in Fig. 2 (a, d, g) the HOMO/LUMO is uniformly distributed for both Pristine graphene and Pristine graphene/Pyrrolidine structures then became around the HMC groups in case of Pristine graphene/Pyrrolidine/2HMC. For MESP maps Fig. 2 (b, e, h) the contour is natural uniformly distributed for Pristine graphene, then became more reactive corresponding to Pristine graphene/Pyrrolidine is neutral but distributed close to the HMC group. Finally, the IR spectrum show positive IR vibrations as seen in Fig. 2 (c, f, i) is an indication for the occurrence of an optimum structure as it is a good test for the studied structure.

Figure 3 presented the B3LYP/6-31G(d, p) calculated parameters for ATRI, ATRI /Pyrrolidine and ATRI/Pyrrolidine/Benzene/2HMC. Structures indicated the behavior indicated in Fig. 2, with no IR spectrum calculated for ATRI /Pyrrolidine/2HMC.

The paramters for ZHEX, ZHEX/Pyrrolidine and ZHEX/Pyrrolidine/Benzene/2HMC which indicated in Fig. 4 are in good agreement with those in Figs. 2 and 3.

Figure 5 presentd the calculated parameters for ZTRI, ZTRI /Pyrrolidine and ZTRI /Pyrrolidine/2HMC. The HOMO/LUMO indicated in Fig. 5 (a, d, g) show uniform distribuation along the edge corresponding to ZTRI /Pyrrolidine and ZTRI /Pyrrolidine/2HMC. While MESP maps in Fig. 5 (b, e, h) are natural but affected by Pyrrolidine/2HMC as previously indicated for other structures. ZTRI /Pyrrolidine/2HMC shows no IR spectrum at B3LYP/6–31 g(d, p).

**Table 1:** DFT: B3LYP/6-31G(d, p) calculated total dipole moment TDM and HOMO/LUMO energy gap ∆E and molecular dimension as Å for the studied model molecules.

Structure	TDM, Debye	∆E, eV	Molecular dimension Å
Pristine	0.0000	4.0344	
Pristine/Pyrrolidine	3.5179	3.4583	
Pristine/Pyrrolidine/Benzene/2HMC	5.5570	2.8090	9.279
ATRI	0.0009	3.1930	
ATRI/Pyrrolidine	4.6908	2.8131	
ATRI/Pyrrolidine/Benzene/2HMC	4.5956	2.4651	17.884
ZHEX	0.0000	2.8210	
ZHEX/Pyrrolidine	4.0672	2.6896	
ZHEX/Pyrrolidine/Benzene/2HMC	3.8286	2.4825	13.754
ZTRI	0.0021	0.3216	
ZTRI/Pyrrolidine	7.6579	0.2884	
ZTRI/Pyrrolidine/Benzene/2HMC	6.6474	0.2844	14.183
AHEX	0.0000	3.5892	
AHEX/Pyrrolidine	3.8230	2.9821	
AHEX/Pyrrolidine/Benzene/2HMC	5.2611	2.2950	14.165

### Density of states DOS analyses

**Fig. 7:**
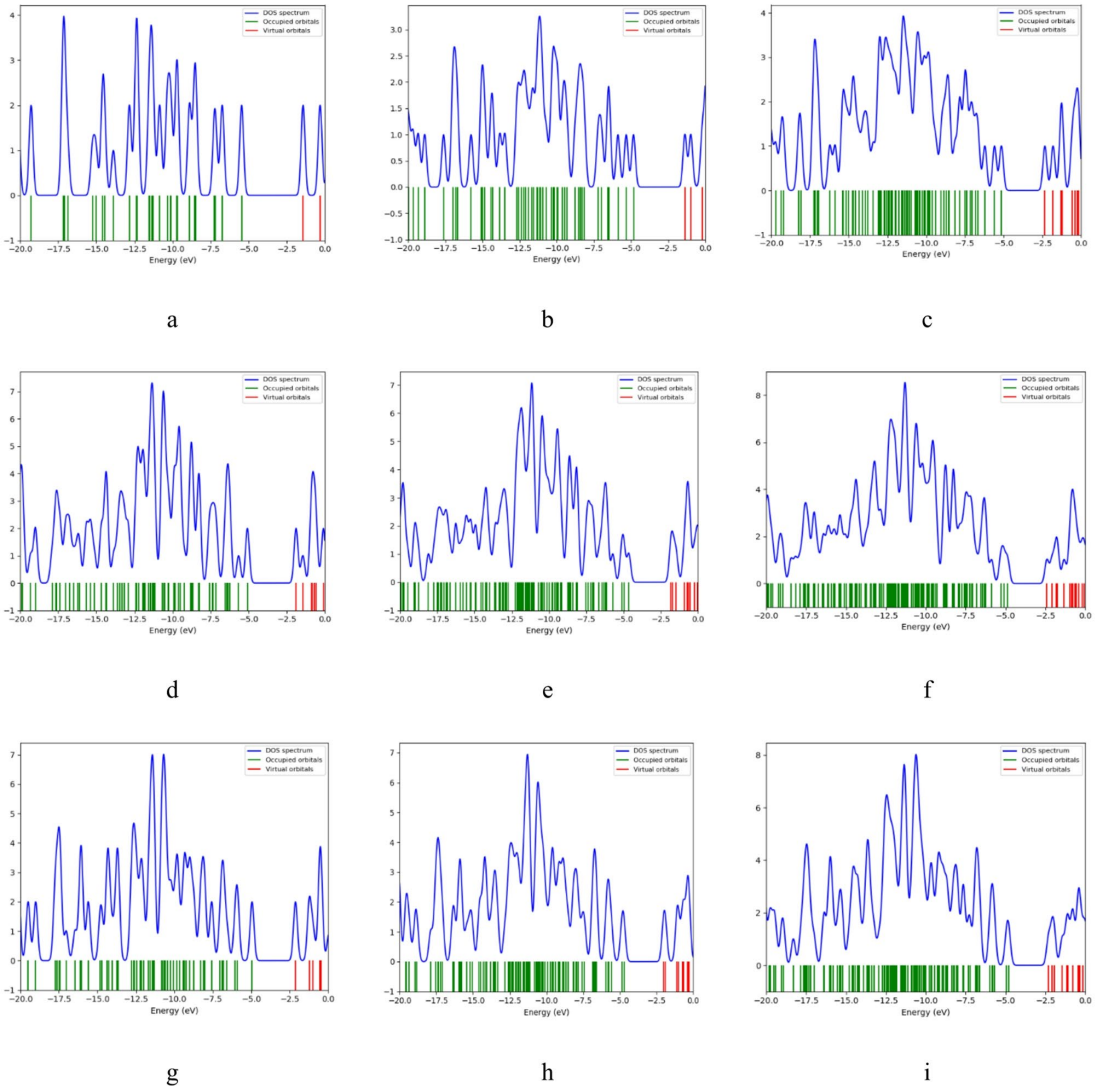

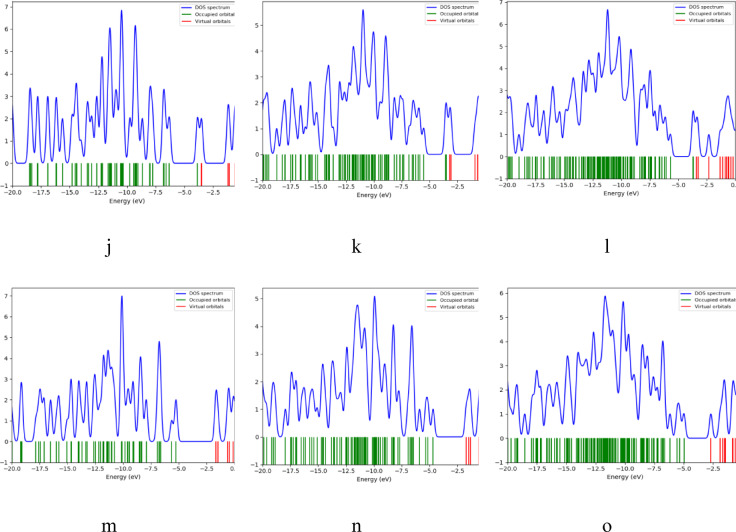
DFT: B3LYP/6-31G(d, p) calculated Density of States for the studied structures whereas; (**a**) Pristine, (**b**) Pristine/Pyrrolidine, (**c**) Pristine/Pyrrolidine/2HMC, (**d**) ATRI, (**e**) ATRI /Pyrrolidine, (**f**) ATRI/Pyrrolidine/Benzene/2HMC, (**g**) ZHEX, (**h**) ZHEX/Pyrrolidine, (**i**) ZHEX/Pyrrolidine/Benzene/2HMC, (**j**) ZTRI, (**k**) ZTRI /Pyrrolidine, (**l**) ZTRI/Pyrrolidine/ Benzene/2HMC, (**m**) AHEX, (**n**) AHEX /Pyrrolidine and (**o**) AHEX/Pyrrolidine/Benzene/2HMC.

More insight into the orbitals could be achieved with the density of states (DOS). DOS is considered as a key concept in molecular science, as it quantifies the number of electronic states available at each energy level. It provides insight into how many states are available for electrons to occupy at each energy level. The DOS plots for the studied structures were calculated at the DFT: B3LYP/6-31G(d, p) level of theory. These plots reveal a clear separation between occupied orbitals and virtual orbitals for all structures, divided by the energy band gap. As illustrated in Fig. 7, for graphene the addition of Pyrrolidine causes the band gap to shrink, and it decreases further with the inclusion of 2HMC, accompanied by an increase in available virtual states. For GQD ATRI, the band gap remains largely unchanged after the addition of Pyrrolidine but decreases upon the incorporation of 2HMC. In the case of GQD ZHEX, the band gap remains similar; however, both Pyrrolidine and 2HMC lead to an increase in the number of virtual states. For GQD ZTRI, a very small band gap is observed, with the states moving closer to the Fermi level in the ZTRI/Pyrrolidine/2HMC structure. Finally, for GQD AHEX, the band gap shrinks after adding Pyrrolidine and 2HMC, with more virtual states becoming available.

### Calculated QTAIM topology

**Fig. 8:**
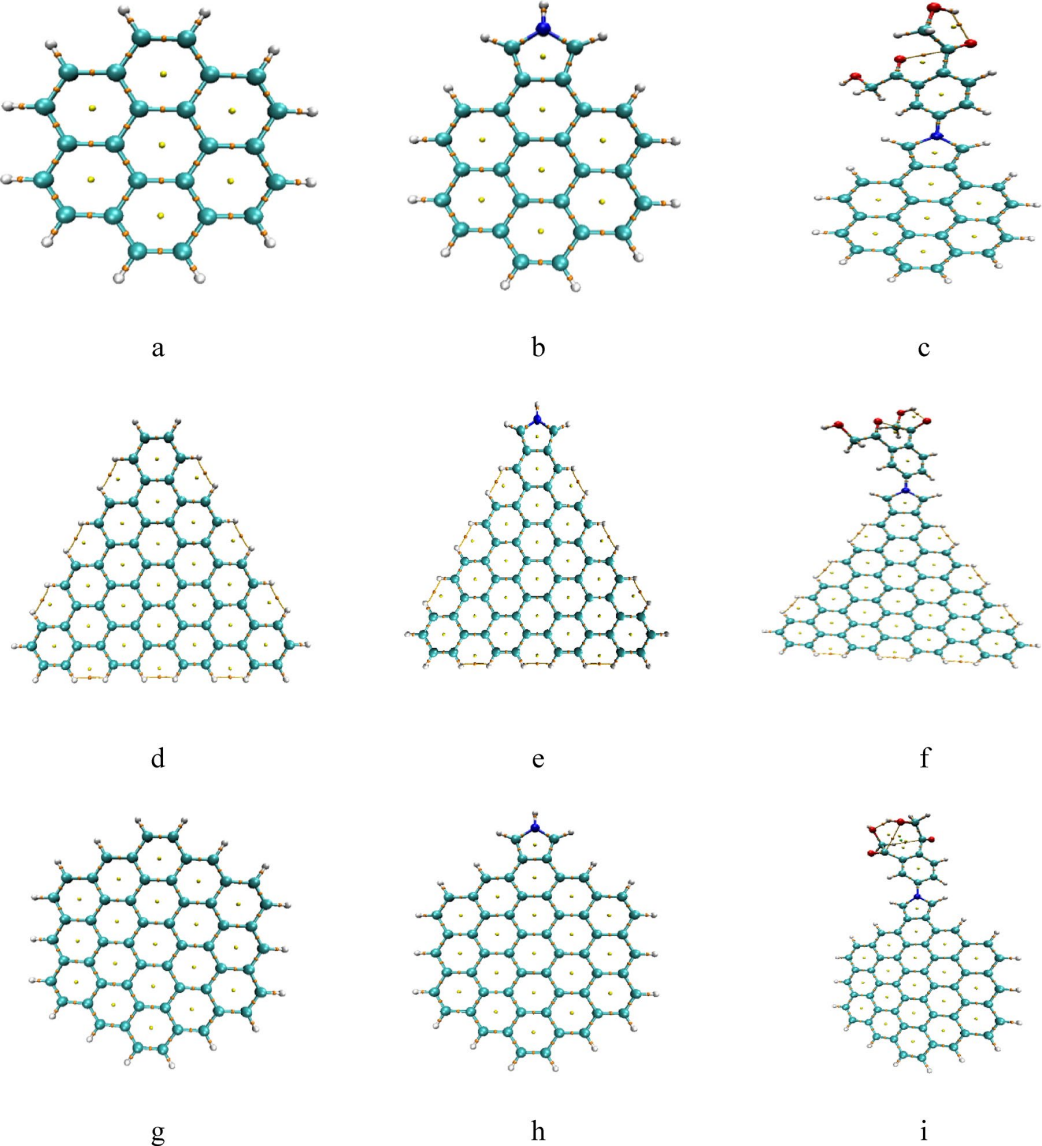

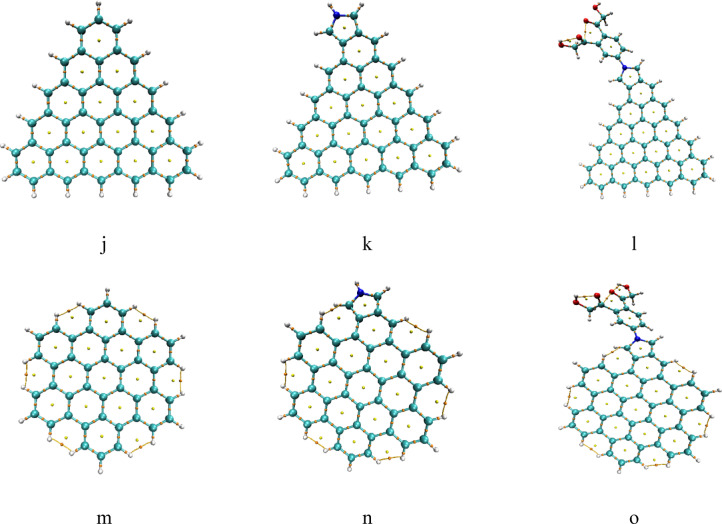
DFT: B3LYP/6-31G(d, p) calculated QTAIM for the studied structures whereas; (**a**) Pristine, (**b**) Pristine/ Pyrrolidine, (**c**) Pristine/ Pyrrolidine /2HMC, (**d**) ATRI, (**e**) ATRI/ Pyrrolidine, (**f**) ATRI/Pyrrolidine/Benzene/2HMC, (**g**) ZHEX, (**h**) ZHEX/Pyrrolidine, (**i**) ZHEX/ Pyrrolidine /2HMC, (**j**) ZTRI, (**k**) ZTRI/Pyrrolidine, (**l**)ZTRI/Pyrrolidine/Benzene/2HMC, (**m**) AHEX, (**n**) AHEX/Pyrrolidine and (**o**) AHEX/Pyrrolidine/Benzene/2HMC. Colour coding: Green: Carbon (C); White: Hydrogen (H); Red: Oxygen (O); Blue: Nitrogen (N).

Although the most active structure (ZTRI/Pyrrolidine/ Benzene/2HMC) was selected based on both TDM and ∆E. Another test will be also used to judge the reactivity in terms of the stability of the studied modified GQDs interacted with aspartic acids. To evaluate stability for all structures, Bader’s quantum theory of atoms in molecules (QTAIM) analysis was conducted to access the topology and stability for the studied GQDs structures and with Pyrrolidine and Pyrrolidine /2HMC. QTAIM allows us to map the distribution of electron density through the molecular system, pinpointing critical points and bond paths^52,53^. In Fig. 8 the plots derived from QTAIM analysis display critical points and bond paths for the structures where the orange balls represent the bond critical points (BCP) and yellow is ring critical points (RCP). The analysis reveals no missing BCPs within the molecular graphs. The presence of all expected BCPs is a significant indicator of the structural integrity and stability of these molecules. Conversely, the absence of a BCP would suggest a weakening or breaking of a bond, implying potential instability. Interestingly, a cage critical point (CCP) appeared for ZHEX/Pyrrolidine/Benzene/2HMC, where the electron density (ρ) is a local minimum in all three spatial directions. Moreover, the presence of BCPs between edge hydrogen atoms in ATRI and AHEX suggests non-negligible interactions between these atoms, potentially implying some degree of additional stabilization. Based on these results, it is clear that all the studied structures are stable.

### Application of ZTRI/pyrrolidine/benzene/2HMC

**Fig. 9:**
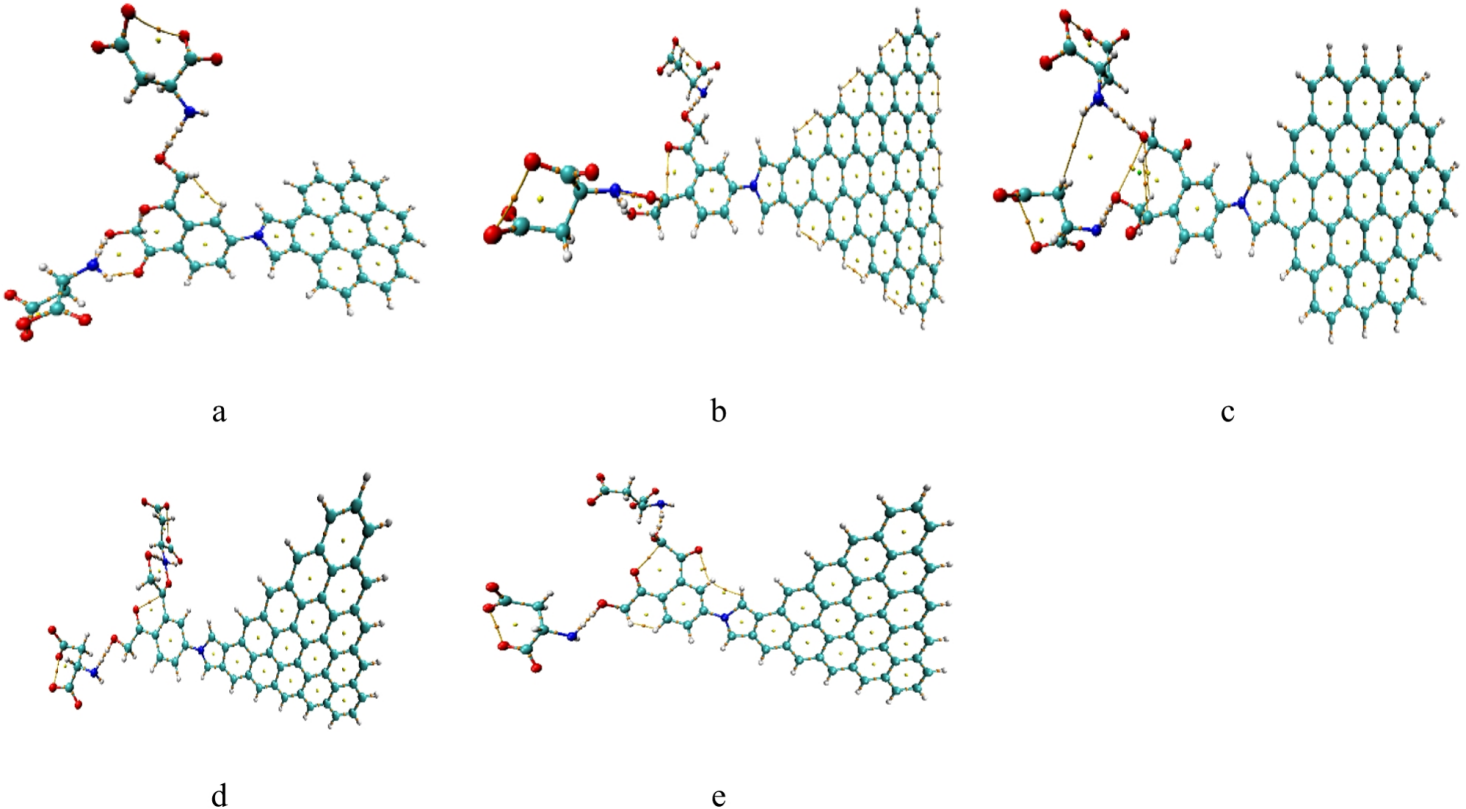
DFT: B3LYP/6-31G(d, p) calculated QTAIM for GQDs/Pyrrolidine/Benzene/2HMC interacted with two aspartic acid through hydrogen bonding whereas (**a**) Pristine; (**b**) ATRI; (**c**) ZHEX; (**d**) ZTRI and (**e**) AHEX. Colour coding: Green: Carbon (C); White: Hydrogen (H); Red: Oxygen (O); Blue: Nitrogen (N).

It was stated earlier that, the amino acids in HIV-1 protease active site are hydrophobic except two aspartic acids namely Asp25 and Asp125, which are hydrophilic^50,51^. the modified graphene qutnum dots with two HMC groups could be tried as HIV-1 protease inhibitors. The possible applications of the studied compunds as HIV-1 protease inhibitors will be investigated via calculation of their possible interaction with the aspartic acid of the HIV-1 protease active sites. Accordingly, each structure of modified graphene quantum dots is supposed to interact with two aspartic acids throughout the H-bonding of the HMC group as indicated in Fig. 9a, b, c, d and e. Based on electron density values ρ and the lablacian ∇²ρ, modified ZTRI after optimization the two weak bonds with the 2 Methoxy exhibt partial covalent character evident by ρ > 0.2 a.u. and ∇²ρ < 0, while modified Pristine, ATRI, ZHEX and AHEX interacted weakly with the 2 Methoxy. The modified ZITRI QTTAIM results in agreement with energy gap, TDM and DOS.

**Fig. 10:**
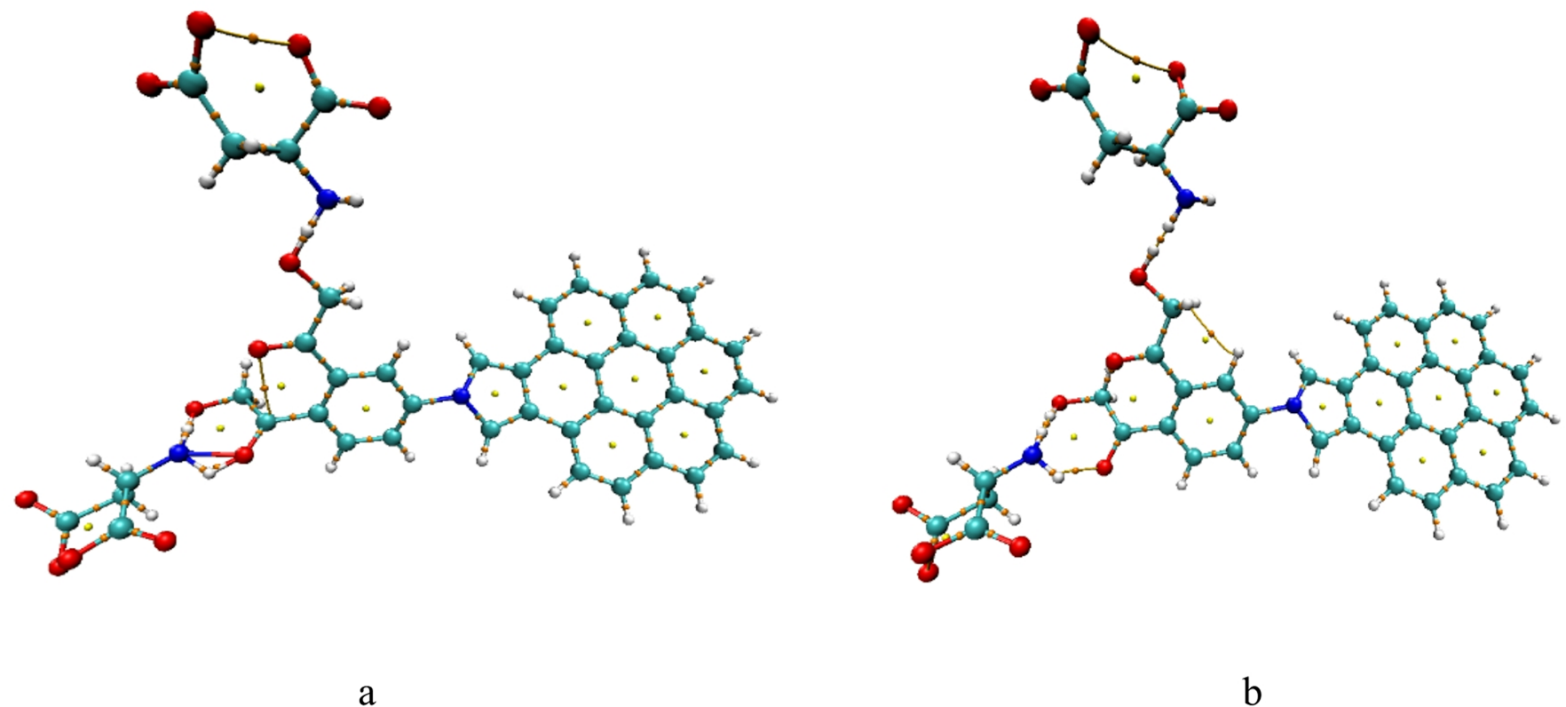
DFT: B3LYP/6-31G(d, p) calculated QTAIM for Pristene/Pyrrolidine/Benzene/2HMC interacted with two aspartic acid whereas (**a**) strongly bonded and (**b**) weakly bonded. Colour coding: Green: Carbon (C); White: Hydrogen (H); Red: Oxygen (O); Blue: Nitrogen (N).

We investigated GQDs/Pyrrolidine/Benzene/2HMC interaction with two aspartic acid through hydrogen bonding following complex and weak interaction, QTAIM analysis was performed to quantify bond strengths and assess changes due to different initial settings. Figure 10 display the modified pristine interaction with two aspartic acid where Figure 10a formed a complex interaction and the same composite in Figure 10b, interacted weakly at the same sites. The modifed pristine retains its covalent bonds when formed complex bond, and remained weakly bonded in second composite, as indicated by electron density values of ρ < 0.2 a.u. and positive values of ∇²ρ. Both composites are stable indicated by their clear electron paths and critical points. Additionally, the complex structure exhibits more newly formed physical and covalent bonds.

## Conclusion

Five types for graphene quantum dots were modified with Pyrrolidine/benzene then further modified with two hydroxymethylcarbonyl (HMC) groups. Two geometric interaction positions, including strongly bonded and weakly bonded states, were proposed to determine the location of the minimum energy complex. Bond strengths and topological stability were assessed using QTAIM analysis, which enabled the identification of authentic physical interactions while ensuring that the results were not affected by Basis Set Superposition Error (BSSE).

Results indicated that, the molecular dimension varies from 9.279 Å corresponding to Pristine/Pyrrolidine/Benzene/2HMC structure up to 17.884 Å corresponding to ATRI/Pyrrolidine/Benzene/2HMC structure. The ZTRI/Pyrrolidine/2HMC model show higher TDM (6.6474 Debye) with lower ∆E (0.2800 eV), it could be more reactive with the surrounding molecules.

The HOMO/LUMO is uniformly distributed for both Pristine graphene and Pristine graphene/Pyrrolidine sructures then became around the HMC groups in case of Pristine graphene/Pyrrolidine/2HMC.

For MESP maps the contour is uniformly distributed for Pristine graphene, then became more reactive corresponding to Pristine graphene/Pyrrolidine is netural but distributed close to the HMC group.

The Total Dipole Moment results show that it depends on the GQD cluster geometry and edge termination because zigzag triangular cuts produce the highest base reactivity. The HMC functional groups establish the minimum energy complex configuration through their specific hydrogen bonding sites that interact with aspartic acid units according to MESP and QTAIM analyses. It is clear that, the reactivity of the studied structures is not a uniform property but is specifically dictated by the topology (armchair vs. zigzag) and the spatial arrangement of the functional groups.

In terms the calculated IR four type of GQDs were optimum, while QTAIM confirmed that all the studied GQDs are stable. The modified GQDs show the ability to form hydrogen bonding with two aspartic acids throughout the carboxyle group of Aspartic acid via hydrogen bonding of the two HMC groups. The cavity of HIV-1 protease active site is about 10 Å in diameter as stated earlier^54, 55^. 

In terms the protease active site diameter the Pristine/Pyrrolidine/Benzene/2HMC show molecular dimension about 9.279 Å which fits into the cavity of the protease active site. While, in terms the reactivity the ZTRI/Pyrrolidine/2HMC show the most reactive sturcture.

The interaction is storngly covalent even when the structure is supposed to interact following complex interaction between modified GQDs and the two aspartic acids.

It was also stated that, the amino acids in HIV-1 protease active site are hydrophobic except two aspartic acids namelyAsp25 and Asp125, are hydrophilic^56^. As far as the studied structures forming hydrogen bonding with two aspartic acids. In terms the QTAIM analyses, the modified GQDs form stable structures and provide strong van der Waals interaction between modified GQDs and HIV-1 protease active sites. This paves the way into application of the studied modified GQDs as effectibe HIV-1 protease inhibitors. Simply, the overall findings will provide fundamental insights into the structure-activity relationship of GQD-based inhibitors, guiding the design of highly effective and specific carbon nanomaterials for anti-HIV-1 drug development.

## Data Availability

The data will be available upon request. Contact Medhat A. Ibrahim, Email: Medhat.AbdElkhalk@bnu.edu.eg.
